# Comparative Evaluation of Large Language Models in Explaining Radiology Reports: Expert Assessment of Readability, Understandability, and Communication Features

**DOI:** 10.1186/s13244-025-02121-3

**Published:** 2025-10-29

**Authors:** Ahmet Bozer, Yeliz Pekçevik

**Affiliations:** 1 Department of Radiology, Ministry of Health Izmir City Hospital, Izmir, Turkey; 2 Department of Radiology, Izmir Faculty of Medicine, University of Health Sciences, Izmir, Turkey

**Keywords:** Radiology reports, Artificial intelligence, Large language models (LLMs), Patient communication, Medical correctness

## Abstract

**Objectives:**

To compare understandability, readability, and communication characteristics of radiology report explanations generated by three freely accessible large language models—ChatGPT, Gemini, and Copilot—based on a standardized prompt, as assessed by expert reviewers.

**Materials and methods:**

In this retrospective single-center study, 100 anonymized radiology reports were randomly selected from five subspecialties. Each report was submitted to ChatGPT (GPT-3.5), Gemini, and Copilot between May 23 and May 30, 2025, using the prompt, “Can you explain my radiology report?”. Responses were evaluated for medical correctness on a 3-point scale (0–2), understandability using the patient education materials assessment tool for understandability (PEMAT-U), and readability using Flesch Reading Ease (FRE), Automated Readability Index (ARI), and Gunning Fog Index (GFI). Communicative features—including uncertainty language, patient guidance, and clinical suggestions—were also assessed. Anxiety-inducing potential was rated on a 3-point Likert scale.

**Results:**

All models demonstrated high medical correctness (mean: 1.97 ± 0.17/2). ChatGPT produced the most readable (FRE: 60.33 ± 3.65; ARI: 9.66 ± 1.01; GFI: 9.1 ± 1.04) and understandable (PEMAT-U: 89.58 ± 3.90%) responses (*p* < 0.01). Copilot included the most uncertainty language (1.62 ± 0.62) and clinical suggestions (1.69 ± 0.60), while Gemini provided the strongest patient guidance (1.62 ± 0.58) (all *p* < 0.01). Only Copilot showed subspecialty-related variation in readability (GFI; *p* = 0.048). Anxiety potential was low across all models (mean: 0.07 ± 0.33).

**Conclusion:**

ChatGPT offered superior readability and understandability. Copilot delivered more clinical detail with cautious language, while Gemini emphasized patient-centered guidance. These differences support context-specific use of language models in radiology communication.

**Critical relevance statement:**

This study shows that freely accessible large language models produce radiology report explanations with varying levels of readability, understandability, and communication quality. Expert-based findings may help inform future strategies to optimize patient-facing applications of AI in radiological communication.

**Key Points:**

- This study compared how freely available AI chatbots respond to patient queries about radiology reports.
- Significant differences were found in understandability, readability, patient guidance, and use of uncertainty or clinical suggestions.
- Findings support context-specific use of AI tools to improve radiology communication and patient understanding.

**Graphical Abstract:**

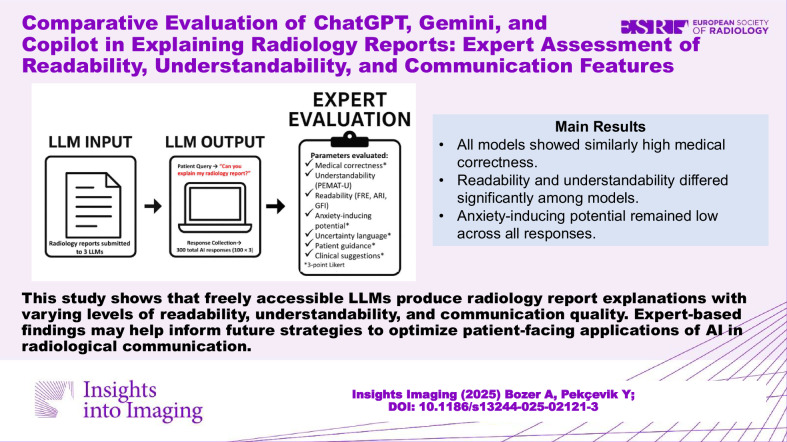

## Introduction

The integration of artificial intelligence (AI) into healthcare has seen unprecedented growth in recent years, particularly with the widespread adoption of large language models (LLMs). Tools such as OpenAI’s ChatGPT, Google Gemini, and Microsoft Copilot have introduced new ways for patients to access and understand complex medical information, including radiology reports—an area known for its high technicality and patient comprehension challenges [1].

Radiology reports are crucial clinical documents that guide medical decision-making, yet they often contain domain-specific terminology and dense phrasing, making them difficult for patients to interpret [2]. Misinterpretation of radiology reports may potentially contribute to patient anxiety, confusion, or misinformed decisions; however, such implications remain hypothetical given the limited patient-based evidence. LLMs, capable of generating natural language explanations, offer a potential bridge between radiologists and patients by translating technical findings into patient-friendly language [1, 3, 4].

Recent studies underscore the growing role of LLMs in radiology. For example, ChatGPT has shown promise in tasks such as generating impressions from reports [5], assisting with differential diagnosis based on imaging patterns [6], and determining appropriate imaging protocols from clinician requests [7]. Additionally, LLMs have been explored as educational tools, with ChatGPT nearly passing a radiology board-style examination even without domain-specific training [8].

Of particular relevance, emerging research has assessed LLMs in simplifying radiology reports for laypersons [9]. For example, Lyu et al [10] demonstrated the feasibility of using ChatGPT to translate radiology reports into plain language for patients and providers. In one exploratory study, radiologists rated ChatGPT-generated patient summaries as factually accurate, sufficiently complete, and generally safe; however, instances of misinformation and ambiguity were also noted, highlighting the need for expert oversight [11]. Similarly, Butler et al [12] showed that AI-generated explanations significantly improved the readability of orthopedic radiology reports, achieving readability levels below the 8th-grade level with minimal factual inaccuracies. Gupta et al [13] recently demonstrated that LLM-simplified radiology reports improved oncology patients’ understanding and confidence compared to original reports.

Despite recent advances in generative AI, a critical gap remains in the literature: few studies have systematically compared multiple LLMs in how they communicate radiological findings to patients, particularly beyond diagnostic accuracy to include key communicative dimensions such as understandability, readability, uncertainty language, patient guidance, clinical suggestions, and anxiety-inducing potential [9, 14]. To address this gap, the present study evaluates responses generated by three freely accessible LLMs—ChatGPT (GPT-3.5), Google Gemini, and Microsoft Copilot—to a standardized patient query regarding a radiology report, aiming to assess their appropriateness for patient education and radiological communication.

## Materials and methods

### Patient report selection

This retrospective study was initiated after obtaining approval from the Non-Interventional Research Ethics Committee of the Ministry of Health Izmir City Hospital (decision no: 2025/200). Given the retrospective design and the use of anonymized data, the requirement for written informed consent was waived by the ethics committee.

**Fig. 1:**
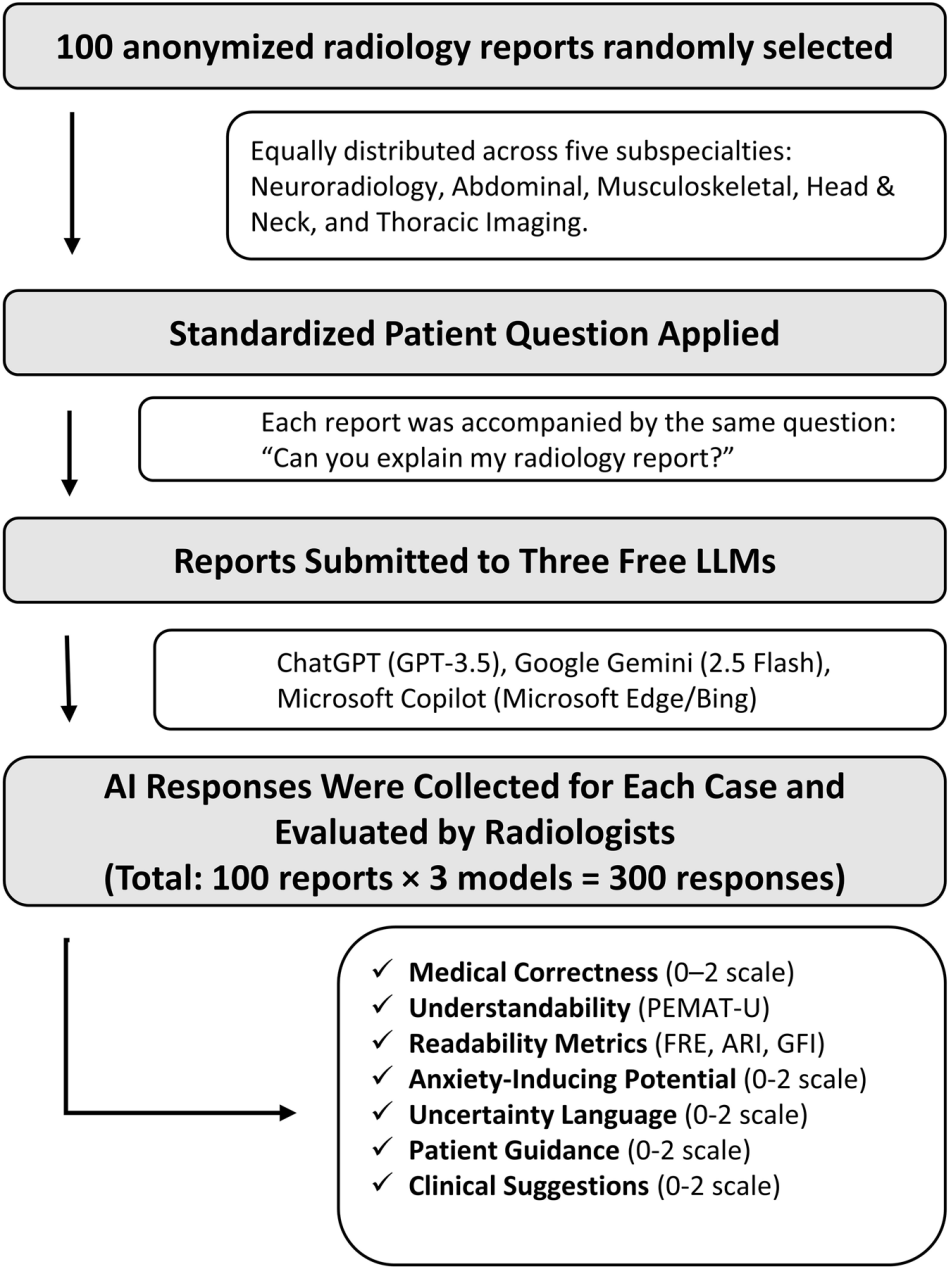
Study flowchart showing LLMs' evaluation of radiology reports

This retrospective study included 100 anonymized radiology reports, randomly selected from clinical cases performed between January 20/24 and January 2025 at a tertiary hospital. The dataset comprised CT and magnetic resonance imaging (MRI) examinations, included in approximately equal proportions, and the reports were evenly distributed across five radiological subspecialties, including neuroradiology, abdominal imaging, musculoskeletal imaging, head and neck imaging, and cardiothoracic imaging (Fig. 1).

Inclusion criteria were radiology reports of individuals aged over 18 years, obtained for clinical indications, and containing clear diagnostic findings, including benign or age-appropriate variants such as incidental or clinically insignificant findings.

Exclusion criteria were reports not in a structured format, incomplete or insufficient reports, reports from pediatric patients (< 18 years), follow-up or post-treatment monitoring reports, non-diagnostic reports with poor image quality, and fully normal reports without any diagnostic or descriptive findings.

All reports were de-identified prior to analysis to ensure patient privacy.

### AI language models evaluated

Each radiology report was presented with a standardized patient query: “Can you explain my radiology report?” This prompt was submitted to the free versions of three publicly available LLMs: ChatGPT Free (GPT-3.5, OpenAI, May 2025 release), Google Gemini (Gemini 2.5 Flash, free version available via the Gemini app, May 2025), and Microsoft Copilot (GPT-4-based free version integrated in Microsoft Edge/Bing, May 2025). All responses were collected during May 23–30, 2025, with each query submitted once per model without regeneration to reflect a typical user experience. Reports were submitted in unmodified plain-text format, and no additional instructions or clarifications were provided. Default model behavior and settings were used, without activating any paid features or plugins. All generated responses were documented for subsequent evaluation (Supplement Table 1).

Each radiology report was presented with a standardized patient query: “Can you explain my radiology report?” This prompt was submitted to the free versions of three publicly available LLMs: ChatGPT Free (GPT-3.5) by OpenAI, Google Gemini by Google, and Microsoft Copilot by Microsoft (via Edge/Bing). All responses were collected during May 23–30, 2025, with each query submitted once per model without regeneration to reflect a typical user experience. Reports were submitted in unmodified plain-text format, and no additional instructions or clarifications were provided. Default model behavior and settings were used, without activating any paid features or plugins. All generated responses were documented for subsequent evaluation (Supplement Table 1).

This study involved the use of three publicly available AI-based LLMs—ChatGPT (GPT-3.5; OpenAI), Google Gemini (Gemini 2.5 Flash, free version), and Microsoft Copilot (Microsoft, Edge/Bing)—to generate patient-facing explanations of radiology reports based on a standardized prompt. No AI tools were used for data analysis or statistical calculations; statistical analyses were performed using SPSS version 26 (IBM Corp.).

### Assessment protocol

All AI-generated outputs were independently evaluated by two board-certified radiologists with 20 and 8 years of experience in radiology, respectively, through consensus, using the following parameters:

- Medical correctness
  The correctness of each AI-generated response was assessed using a 3-point scale (0 = incorrect, 1 = partially correct, 2 = fully correct). A response was scored as 2 (fully correct) if it accurately reflected the key radiological findings described in the original report.
- Understandability (PEMAT-U)
  Understandability refers to how easily individuals with diverse backgrounds and varying levels of health literacy can comprehend and interpret patient-facing information. To assess this dimension, the study employed the patient education materials assessment tool-understandability (PEMAT-U), a validated instrument developed specifically to evaluate written health education content [15].
  In the context of this study, AI-generated responses were treated as written patient education materials. Among the 17 standard PEMAT-U items, 16 were deemed applicable to the format and content of the responses. Each item was scored as “1” if the criterion was met, “0” if not met, and “N/A” if not applicable, and excluded from the denominator (e.g., Item 13 “The material’s visual aids have clear titles or captions” was excluded as not applicable to text-only responses). The final understandability score was calculated as:
  $${{\mathrm{PEMAT}}}-{{\rm{U}}}\; {{\rm{Score}}}\,( \% )=({{\rm{Total}}}\; {{\rm{number}}}\; {{\rm{of}}}\,^{\prime\prime} 1^{\prime\prime} \,{{\mathrm{scores}}}/16)\times 100$$
  A higher PEMAT-U percentage indicates greater understandability.
  The “Actionability” component of the PEMAT was not assessed, as it includes criteria relevant to visual aids and behavioral instructions that were not applicable to the nature of the responses analyzed. Higher PEMAT-U scores indicate better understandability and enhanced potential for patient comprehension.
- Readability indices
  To objectively evaluate the textual complexity of the AI-generated responses, three established readability indices were calculated: Flesch Reading Ease (FRE), Automated Readability Index (ARI), and Gunning Fog Index (GFI). All readability scores were computed using freely available, web-based tools to ensure consistency and transparency across analyses.
  FRE assesses how easy a text is to read, with the score calculated using the formula:
  $${{\rm{FRE}}}=206.835-(1.015\times {{\rm{ASL}}})-(84.6\times {{\rm{ASW}}})$$
  where ASL is the average sentence length and ASW is the average number of syllables per word. FRE scores range from 0 to 100, with higher scores indicating simpler, more accessible language. A score of 60–70 is typically considered acceptable for a general audience.
  ARI estimates the U.S. grade level required to comprehend the text. The formula is:
  $${{\rm{ARI}}}=4.71\times ({{\rm{characters}}}/{{\rm{words}}})+0.5\times ({{\rm{words}}}/{{\rm{sentences}}})-21.43$$
  Lower ARI values correspond to easier readability, with scores aligned to educational grade levels (e.g., an ARI of 8 implies comprehension by an 8th-grade student).
  GFI estimates the number of years of formal education a person needs to understand the text on first reading. It is calculated as:
  $${{\rm{GFI}}}=0.4\times [({{\rm{words}}}/{{\rm{sentences}}})+100\times ({{\rm{complex\; words}}}/{{\rm{words}}})]$$
  where complex words are defined as those with three or more syllables, excluding proper nouns and familiar jargon. GFI scores of 7–8 are ideal for materials intended for a general audience, while higher values indicate increasing difficulty.
  Readability scores were calculated through publicly available online platforms designed for text analysis [16]. These indices provided a standardized, quantitative assessment of the linguistic accessibility of each AI-generated output.
- Anxiety-inducing potential

The potential of each response to provoke patient anxiety was assessed using a 3-point Likert scale (0 = No, 1 = Partially, 2 = Yes), based on the presence of alarming or emotionally charged language. Responses stating or implying serious conditions without appropriate context or reassurance (e.g., “This indicates brain damage” or “This could be cancer”) were scored higher. Additionally, responses that mentioned serious diagnoses without being explicitly stated in the report or lacked explanatory context (e.g., “The findings may be consistent with a brain tumor”) were also considered to increase anxiety. Direct warnings or urgent calls to action—such as “Consult your physician immediately” or “Urgent medical intervention may be required”—were classified as anxiety-provoking.

#### Communicative content evaluation

Three additional communication dimensions were evaluated on a 3-point scale (0 = No, 1 = Partially, 2 = Yes):

- Uncertainty language, indicating whether the response acknowledged diagnostic ambiguity or limitations (e.g., “These findings may suggest several possibilities; clinical correlation is advised”);
- Patient guidance, assessing the inclusion of informative suggestions for patients, such as follow-up or next steps (e.g., “You should consult your doctor to determine the cause of these findings”; “These imaging findings may require further evaluation. Follow-up imaging or additional tests may be recommended”; “If you are experiencing new symptoms such as headache or visual disturbances, you should consult a healthcare provider”);
- Clinical suggestion, capturing whether the AI attempted to provide diagnostic or treatment-related recommendations (e.g., “These findings may indicate microvascular ischemia, commonly managed by controlling blood pressure,” or “An antibiotic may be needed if this represents an infection”; “This may reflect demyelinating disease; neurologic evaluation and possibly corticosteroid treatment may be required”; “In the context of cardiovascular risk factors, these findings should prompt optimization of blood pressure and lipid control”).

Both radiologists were already familiar with the readability indices (FRE, ARI, and GFI) through prior academic work and scientific reporting. To ensure consistency, a short calibration session on the application of the PEMAT-U tool was conducted before the assessments. No further formal training was required.

### Statistical analysis

All statistical analyses were conducted using SPSS version 26 (IBM Corp.). Due to the non-normal distribution of continuous variables (assessed visually and/or via normality tests), non-parametric methods were applied. Group differences among the three AI models were evaluated using the Kruskal–Wallis *H*-test for each outcome variable. When significant, post hoc pairwise comparisons were performed using two-tailed Mann–Whitney *U*-tests with Bonferroni correction to control for multiple comparisons. A *p*-value < 0.05 was considered statistically significant. Continuous data are reported as mean ± standard deviation (SD), and categorical variables as frequencies and percentages.

## Results

The medical correctness of all three AI models was identical, with a mean score of 1.97 ± 0.17 out of 2, and no statistically significant difference was observed among them (*p* > 0.05; Table 1).

**Table 1:** Comparison of mean scores across AI models for medical correctness, understandability, readability, and communication characteristics

Metric	ChatGPT (mean ± SD)	Gemini (mean ± SD)	Copilot (mean ± SD)	*p*-value*
Medical Correctness**	1.97 ± 0.17	1.97 ± 0.17	1.97 ± 0.17	> 0.05
Understandability Score (PEMAT-U) (%)	89.58 ± 3.90	84.54 ± 3.94	73.70 ± 4.35	*< 0.001*
FRE	60.33 ± 3.65	49.06 ± 3.25	45.62 ± 2.50	*< 0.001*
ARI	9.66 ± 1.01	11.55 ± 0.92	12.50 ± 0.99	*< 0.001*
GFI	9.10 ± 1.04	11.09 ± 1.05	11.38 ± 0.96	*< 0.001*
Anxiety-inducing potential**	0.07 ± 0.33	0.07 ± 0.33	0.07 ± 0.33	> 0.05
Uncertainty language**	0.88 ± 0.69	1.18 ± 0.80	1.62 ± 0.62	*< 0.001*
Patient guidance**	1.02 ± 0.72	1.62 ± 0.58	1.47 ± 0.72	*< 0.001*
Clinical suggestion**	1.09 ± 0.81	1.50 ± 0.66	1.69 ± 0.60	*< 0.001*

Italic values mean statistically significant

^*^ Kruskal–Wallis *p*-values; *p* < 0.05 = significant

^**^ 3-point Likert scale (0 = No, 1 = Partially, 2 = Yes)

In contrast, significant differences emerged in understandability, readability, and communicative characteristics. ChatGPT achieved the highest understandability score (PEMAT-U: 89.58 ± 3.90%), followed by Gemini (84.54 ± 3.94%) and Copilot (73.70 ± 4.35%), with all pairwise comparisons yielding statistically significant results (*p* < 0.01; Tables 1 and 2).

**Table 2:** Post Hoc pairwise comparisons of AI models across understandability, readability, and communication metrics

Metric	Comparison	Corrected *p*-value^*^
Understandability score (PEMAT-U)	chatgpt vs gemini	*p* < *0.001*
Understandability score (PEMAT-U)	Chatgpt vs copilot	*p* < *0.001*
Understandability score (PEMAT-U)	Gemini vs Copilot	*p* < *0.001*
FRE	chatgpt vs gemini	*p* < *0.001*
FRE	chatgpt vs copilot	*p* < *0.001*
FRE	gemini vs copilot	*p* < *0.001*
ARI	chatgpt vs gemini	*p* < *0.001*
ARI	chatgpt vs copilot	*p* < *0.001*
ARI	gemini vs copilot	*p* < *0.001*
GFI	chatgpt vs gemini	*p* < *0.001*
GFI	chatgpt vs copilot	*p* < *0.001*
GFI	gemini vs copilot	p = 0.128
Uncertainty language	chatgpt vs gemini	*p* = *0.014*
Uncertainty language	chatgpt vs copilot	*p* < *0.001*
Uncertainty language	gemini vs copilot	*p* < *0.001*
Patient guidance	chatgpt vs gemini	*p* < *0.001*
Patient guidance	chatgpt vs copilot	*p* < *0.001*
Patient guidance	gemini vs copilot	*p* = 0.547
Clinical suggestion	chatgpt vs gemini	*p* = *0.001*
Clinical suggestion	chatgpt vs copilot	*p* < *0.001*
Clinical suggestion	gemini vs copilot	*p* = *0.047*

Italic values mean statistically significant

^*^ Bonferroni-corrected *p*-values from Mann–Whitney *U*-test; *p* < 0.05 is significant

Readability metrics also favored ChatGPT, which achieved the highest FRE (60.33 ± 3.65) and the lowest ARI (9.66 ± 1.01) and GFI (9.10 ± 1.04), reflecting simpler sentence structure and vocabulary (Table 1). In contrast, Copilot produced more complex responses, as reflected in its FRE (45.62 ± 2.50), ARI (12.50 ± 0.99), and GFI (11.38 ± 0.96) scores (Table 1).

Among all evaluated metrics, only the GFI scores of Copilot showed significant variation across radiology subspecialties (*p* = 0.048; Table 3). The highest GFI values were observed in neuroradiology reports (11.84 ± 1.04), indicating greater linguistic complexity compared to musculoskeletal imaging (10.92 ± 0.77; Table 4).

**Table 3:** Comparison of AI language model outputs across radiology subspecialties

Model	Variable	Kruskal–Wallis *H*-test	*p*-value^*^
ChatGPT	Medical correctness	5.443	0.245
ChatGPT	PEMAT-U	5.523	0.238
ChatGPT	FRE	2.356	0.671
ChatGPT	ARI	5.693	0.223
ChatGPT	GFI	2.033	0.730
ChatGPT	Anxiety	4.208	0.379
ChatGPT	Uncertainty language	4.126	0.389
ChatGPT	Patient guidance	0.283	0.991
ChatGPT	Clinical suggestion	0.5	0.973
Gemini	Medical correctness	5.443	0.245
Gemini	PEMAT-U	4.177	0.383
Gemini	FRE	4.488	0.344
Gemini	ARI	2.723	0.605
Gemini	GFI	1.307	0.860
Gemini	Anxiety	4.208	0.379
Gemini	Uncertainty language	2.231	0.693
Gemini	Patient guidance	3.073	0.546
Gemini	Clinical suggestion	1.042	0.904
Copilot	Medical correctness	5.443	0.245
Copilot	PEMAT-U	8.986	0.061
Copilot	FRE	3.048	0.550
Copilot	ARI	0.679	0.954
Copilot	GFI	9.587	*0.048*
Copilot	Anxiety	4.208	0.379
Copilot	Uncertainty language	9.379	0.052
Copilot	Patient guidance	6.121	0.190
Copilot	Clinical suggestion	9.020	0.061

Italic values mean statistically significant

^*^ Kruskal–Wallis *p*-values across subspecialties; *p* < 0.05 = significant

**Table 4:** GFI scores (mean ± SD) of copilot responses by radiology subspecialty

Radiology subspecialty	Mean ± SD of Copilot GFI scores
Abdominal	11.24 ± 1.03
Head and neck	11.56 ± 0.76
Musculoskeletal	10.92 ± 0.77
Neuroradiology	11.84 ± 1.04
Cardiothoracic	11.39 ± 0.97

Anxiety-inducing potential was uniformly low across all three models (0.07 ± 0.33, *p* > 0.05; Table 1). However, Copilot demonstrated the highest use of uncertainty language (1.62 ± 0.62) and clinical suggestions (1.69 ± 0.60), followed by Gemini and ChatGPT, with all differences being statistically significant (*p* < 0.01; Tables 1 and 2). Patient guidance was most frequently observed in Gemini responses (1.62 ± 0.58), while ChatGPT provided significantly less guidance (1.02 ± 0.72, *p* < 0.01 for all comparisons; Table 2).

Overall, ChatGPT outperformed the others in terms of understandability and readability, whereas Copilot offered the most interpretive content, albeit through more complex and less accessible language.

## Discussion

This study offers a structured comparison of three free LLMs (ChatGPT, Gemini, and Copilot) in explaining radiology reports to patients. Although there are differences in terms of understandability, readability, and communicative characteristics, no significant difference was found in medical correctness; all models provided largely correct and consistent outputs. Amin et al [9] found that ChatGPT-3.5 and Bing scored higher than Bard in factual accuracy and completeness when simplifying 150 radiology reports (*p* < 0.05). Infante et al [17] reported that ChatGPT-4 showed the highest diagnostic agreement (κ = 0.645) in emergency CT reports, outperforming Perplexity and Bard (κ = 0.392). Nakaura et al [18] noted that GPT-4 outputs closely resembled radiologist reports but warned against relying on them without expert validation due to hallucination risk. Maintaining a balance between clarity and clinical reliability remains essential.

Among the evaluated LLMs, ChatGPT showed the best performance in both understandability (PEMAT-U: 89.58 ± 3.90%) and readability (FRE = 60.33 ± 3.65; lowest ARI and GFI scores). This suggests its strong potential to produce patient-friendly responses. Traditional radiology reports are often hard to understand; Martin-Carreras et al [2] noted they average a 13th-grade reading level. In contrast, ChatGPT’s FRE score in our study falls within the recommended range for public health materials. Li et al [19] also showed that ChatGPT significantly improved readability, raising the FRE score from 38.0 to 83.5 and reducing the grade level from 10th to 6th with a patient-focused prompt. However, despite its clarity, ChatGPT offered limited interpretative content, scoring lowest in both patient guidance (1.02 ± 0.72) and clinical suggestion (1.09 ± 0.81). It also rarely used uncertainty language—something Copilot used more frequently. These findings highlight ChatGPT’s strength in accessibility but point to its shortcomings in delivering more nuanced and clinically supportive responses.

Other comparative studies also support our findings. Amin et al [9] evaluated four LLMs (ChatGPT-3.5, GPT-4, Bard, Bing) and found that GPT-4 and Bard produced the most readable summaries, while ChatGPT-3.5 generated the shortest and most clinically accurate responses, receiving the highest ratings for patient communication. Similarly, Doshi et al [20] confirmed that all models improved readability and reduced reading level when prompted with patient-centered queries. Tepe et al [21], in their study evaluating thirty radiology reports, found that all AI models (ChatGPT-4, Google Bard, and Microsoft Copilot) effectively simplified medical terminology into more accessible language. Bard achieved the highest readability scores, while ChatGPT-4 and Bard stood out with understandability scores exceeding 70% (*p* < 0.01). These results highlight the role of prompt design in enhancing LLM communication.

In contrast, Copilot often produced longer and more complex outputs, with higher GFI and ARI scores. This aligns with Amin et al [9], who noted that Bing-based models included excessive technical detail that limited clarity. Notably, our study found Copilot provided the most interpretative content, often including clinical suggestions and uncertainty language. While this may resemble a “doctor-like” tone, it could also increase anxiety if not well-framed. Balancing informativeness with emotional clarity is therefore essential.

In the subspecialty-based analysis, only Copilot showed a statistically significant difference in GFI scores (Table 4). The neuroradiology reports generated by Copilot exhibited the highest linguistic complexity. This suggests that the model’s output may be sensitive to the technical density of the original report. In the future, subspecialty-specific fine-tuning of language models could help produce more appropriate and tailored content.

Notably, all models exhibited a generally low anxiety-inducing potential, although this was assessed by expert reviewers rather than patients. This diverges slightly from concerns raised by Amin et al [14], who cautioned that overly technical or ambiguous language from LLMs may elicit patient anxiety. Future studies incorporating direct patient feedback will be essential to validate these observations.

This study has several limitations. Only written texts in English were evaluated; visual or audio outputs were not analyzed within this scope. Additionally, only the freely accessible versions of the LLMs were tested, as they are the most commonly available to the general public. Since these models are continuously updated, future or premium versions may yield different results. Lastly, all evaluations were conducted by radiologists rather than patients, which may limit the generalizability of the findings to actual patient comprehension and emotional response. While tools like PEMAT-U and readability indices provide standardized metrics, they do not fully capture how patients perceive or emotionally react to the content.

Prompt design is an important factor influencing LLM output. In this study, we used a single standardized prompt (“Can you explain my radiology report?”) to ensure comparability across models. While this approach provides consistency, it does not capture the variability that may arise from alternative or optimized prompt strategies. Given that prompt phrasing can significantly affect the tone, completeness, and relevance of model responses, future studies should explore how different prompt designs may influence communication quality and patient comprehension.

Moreover, while our results highlight promising potential for enhancing radiology communication, LLM-generated explanations must be interpreted within the appropriate clinical and ethical framework. Radiology reports are primarily designed for clinician-to-clinician communication and typically contain technical terminology and implicit diagnostic reasoning, which may limit their accessibility for patients [22]. As patients increasingly gain direct access to their medical records, concerns arise regarding potential misinterpretation, confusion, or anxiety. Although LLMs offer valuable tools for simplification, risks such as oversimplification, loss of nuance, or misrepresentation must be considered [23, 24]. Therefore, future efforts to implement LLM-based patient communication should involve expert oversight and prioritize ethical transparency.

## Conclusion

This study provides an expert-based comparative evaluation of three freely accessible LLMs—ChatGPT, Gemini, and Copilot—in generating simplified explanations of radiology reports. While all models demonstrated consistent outputs in terms of diagnostic content, their communication characteristics varied. ChatGPT generated the most readable and understandable responses based on structured readability tools. Copilot produced more interpretative but linguistically complex outputs, while Gemini offered relatively stronger patient guidance with balanced clarity. These findings underscore the potential of LLMs to support patient-facing radiological communication; however, their application should be guided by specific communication goals and supplemented by expert oversight. Future studies incorporating patient perspectives may help further validate these expert-based findings and inform practical implementation strategies.

## Supplementary information

ELECTRONIC SUPPLEMENTARY MATERIAL

## Data Availability

The datasets generated during and/or analyzed during the current study are not publicly available due to institutional restrictions on data sharing, but they are available from the corresponding author on reasonable request.

## Abbreviations

AI: Artificial intelligence
ARI: Automated readability index
FRE: Flesch reading ease
GFI: Gunning fog index
LLM: Large language mode
PEMAT-U: Patient education materials assessment tool-understandability
SD: Standard deviation
