# Supplementary material for: Comparative Evaluation of Large Language Models in Explaining Radiology Reports: Expert Assessment of Readability, Understandability, and Communication Features
Source: Insights Imaging. 2025 Oct 29;16:232. doi: 10.1186/s13244-025-02121-3 (PMC12572556; doi:10.1186/s13244-025-02121-3)
Supplement: Supplementary file 1 — ELECTRONIC SUPPLEMENTARY MATERIAL [file 13244_2025_2121_MOESM1_ESM.docx]

**Comparative Evaluation of Large Language Models in Explaining Radiology Reports: Expert Assessment of Readability, Understandability, and Communication Features**

**Supplement Table 1**. Example of Responses from Three Large Language Models (LLMs) to a Radiology Report-Based Patient Question

| **Radiology Report** | **ChatGPT (GPT-3.5)** | **Gemini** | **Microsoft Copilot** |
| --- | --- | --- | --- |
| "CLINICAL INFORMATION:** Speech disturbance, weakness  **TECHNIQUE:** Non-contrast axial T1-weighted, axial T2-FLAIR, axial T2 PROPELLER, coronal and sagittal sequences, b0–1000 DWI and ADC, axial T2-GRE; contrast-enhanced axial, coronal, and sagittal T1-weighted sequences; contrast-enhanced axial T2-FLAIR, axial T1 SE, and 3D sagittal T1-weighted sequences  **COMPARISON:** No prior brain MRI examination is available in PACS for comparison.  **FINDINGS:**  **Parenchyma:**  A mass lesion consistent with a high-grade glial tumor is observed in the left basal ganglia, insula, and frontal operculum, extending inferiorly to the cerebral peduncle, medially to the body of the corpus callosum and right cingulate gyrus, and inferomedially to the ependymal region. The lesion includes a necrotic area measuring 23 × 31 × 26 mm at the level of the left basal ganglia that shows contrast enhancement.  There is compression on the left lateral ventricle.  No midline shift or herniation is observed.  No other mass lesion is identified in other parenchymal regions.  No evidence of infarction or hemorrhage is seen.  **Extra-axial collection:** Not observed.  **Ventricular system:** Compression of the left lateral ventricle is noted. No dilatation is seen.  **Major intracranial vascular flow-voids:** Normal.  **Bony structures:** Normal signal characteristics.  **Included orbits:** Normal.  **Paranasal sinuses:** Normal aeration.  **Tympanomastoid cavity:** Normal aeration.  **Conclusion:**  A mass lesion consistent with a high-grade glial tumor is observed in the left basal ganglia, insula, and frontal operculum, extending to the cerebral peduncle, corpus callosum, right cingulate gyrus, and ependymal region, with a necrotic contrast-enhancing component in the left basal ganglia. There is compression on the left lateral ventricle. No midline shift or herniation is observed." | "Absolutely, I can walk you through your MRI report in a clear, easy-to-understand way. Here’s a breakdown of what it says and what it might mean:  🧠 What's the Main Finding?  Your MRI shows a mass lesion (tumor) located in the left side of your brain, specifically in areas that control movement, language, and other key functions:  Left basal ganglia – involved in movement coordination  Insula & frontal operculum – involved in speech and language  It also extends to:  Cerebral peduncle – part of the brainstem that carries movement-related signals  Corpus callosum & right cingulate gyrus – areas involved in communication between brain hemispheres and emotions  Ependymal region – lining of the ventricles (fluid-filled spaces in the brain)  🧬 What Kind of Tumor Might This Be?  The appearance is ""consistent with a high-grade glial tumor.""  This often means:  Glioblastoma or another aggressive (fast-growing) type of brain tumor that arises from glial cells (the support cells of the brain).  There’s a necrotic (dead tissue) area inside the tumor, which often happens with high-grade tumors due to rapid growth outpacing blood supply.  🩻 Other Important Findings:  The tumor is pressing on your left lateral ventricle, which is one of the brain’s fluid-filled cavities — but:  ✅ No signs of midline shift or herniation, meaning the pressure isn't causing dangerous displacement of brain structures.  ❌ No bleeding or stroke (infarction) seen.  ✅ No issues seen in bones, sinuses, eyes, or ears.  🧾 Conclusion (Simplified):  You have a large, aggressive-appearing brain tumor on the left side that involves multiple important areas, including movement and speech centers. It has a central area of tissue death and is pushing on one of the brain’s ventricles, though there’s no current evidence of a life-threatening shift in brain structures.  🧠 Symptoms & Clinical Correlation:  Your reported symptoms of speech disturbance and weakness are very consistent with the location of this tumor, especially in the left hemisphere, which typically controls language and the right side of the body.  Next Steps?  You’ll likely need further evaluation by a neurosurgeon and neuro-oncology team.  A biopsy may be done to confirm the exact tumor type and guide treatment (like surgery, radiation, or chemotherapy).  Would you like help preparing questions for your doctor, or want more info on what treatment paths might look like?" | "This MRI report indicates the presence of a significant brain tumor in the left side of your brain. Here's a breakdown of the findings in simpler terms:  * **Mass Lesion:** The main finding is a mass, which the radiologist believes is most likely a **high-grade glial tumor**. Glial tumors arise from the supportive cells (glia) of the brain. ""High-grade"" generally implies a tumor that is more aggressive and tends to grow and spread more quickly.  * **Location:** The tumor is located in several important areas on the left side of your brain:  * **Left Basal Ganglia:** Involved in motor control, behavior, and emotions.  * **Insula:** Plays a role in consciousness, emotions, and various other functions.  * **Frontal Operculum:** Part of the frontal lobe involved in speech and facial movements.  * **Cerebral Peduncle:** A structure in the midbrain that connects higher brain regions to the brainstem and spinal cord.  * **Body of the Corpus Callosum:** The central part of the band of nerve fibers connecting the left and right hemispheres of the brain.  * **Right Cingulate Gyrus:** An area on the opposite side of the brain involved in emotions, learning, and memory.  * **Ependymal Region:** The lining of the ventricles (fluid-filled spaces) in the brain.  * **Necrotic Area with Contrast Enhancement:** Within the tumor in the left basal ganglia, there's a region (measuring 23 × 31 × 26 mm) that is described as ""necrotic."" This means there's an area of dead tissue within the tumor. This necrotic area also shows ""contrast enhancement"" on the MRI after contrast was given. Contrast enhancement typically indicates areas with a disrupted blood-brain barrier, which is common in tumors and often seen in the more active or aggressive parts of the tumor.  * **Compression of the Left Lateral Ventricle:** The tumor is pressing on the left lateral ventricle, one of the fluid-filled spaces in the brain.  * **No Midline Shift or Herniation:** This is a positive finding. It means the tumor, despite its size, has not caused significant pressure to shift the brain's central structures (midline shift) or push brain tissue into other compartments (herniation), which can be very dangerous.  * **No Other Mass Lesion, Infarction, or Hemorrhage:** No other tumors, areas of stroke (infarction), or bleeding (hemorrhage) were seen in the rest of the brain.  * **Normal Other Structures:** The report indicates that the blood vessels, bony structures, orbits (eye sockets), paranasal sinuses, and tympanomastoid cavity (middle ear and mastoid bone) appear normal.  **In summary, the MRI reveals a large, complex tumor on the left side of your brain that is likely a high-grade glial tumor. It involves several important brain structures, has an area of dead tissue within it that shows signs of active growth or increased blood flow, and is compressing one of the brain's fluid-filled spaces. Fortunately, there is no evidence of brain shift or herniation at this time.**  **Next Steps:**  This is a serious finding that requires immediate attention and further evaluation by a neurologist and likely a neuro-oncologist (a specialist in brain tumors). They will likely:  * **Review the MRI images themselves.**  * **Correlate these findings with your clinical symptoms (speech disturbance and weakness).**  * **Recommend further diagnostic procedures, most likely a biopsy.** A biopsy is essential to definitively determine the type and grade of the tumor.  * **Discuss treatment options based on the tumor type, grade, location, and your overall health.** Treatment options for high-grade glial tumors can include surgery, radiation therapy, chemotherapy, and targeted therapies.  It is crucial to follow up with your doctor as soon as possible to discuss these findings and the next steps in your care." | "Certainly, Your MRI report describes a significant finding—a mass lesion in the brain that is consistent with a high-grade glial tumor. Let me break it down for you in simpler terms:  Key Findings:  Tumor Location and Characteristics:  The tumor is located in the left basal ganglia, insula, and frontal operculum. It also extends to nearby areas like the cerebral peduncle, corpus callosum, right cingulate gyrus, and the ependymal region.  It includes a necrotic area (a region of dead tissue) measuring 23 × 31 × 26 mm, which shows contrast enhancement. This means the tumor is actively growing and has areas of tissue breakdown.  Impact on Brain Structures:  The tumor is compressing the left lateral ventricle, which is a fluid-filled space in the brain. However, there is no midline shift (displacement of brain structures) or herniation (serious pressure-related complications), which is a positive sign.  No Additional Issues:  No other tumors, strokes, or bleeding were detected.  Other structures like the paranasal sinuses, orbits, and bony structures appear normal.  What Does This Mean?  A high-grade glial tumor is a type of brain tumor that originates from glial cells, which support and protect nerve cells. These tumors are often aggressive and require prompt medical attention.  The compression of the ventricle could potentially contribute to symptoms like headaches, speech disturbances, or weakness, as mentioned in the clinical information.  Next Steps:  Your healthcare provider may recommend further tests, such as a biopsy, to confirm the tumor type and grade.  Treatment options might include surgery, radiation therapy, and/or chemotherapy, depending on the tumor's characteristics and your overall health.  I understand this can be overwhelming, and I'm here to help clarify anything further. How are you feeling about this information?" |
